# Mycorrhizae Enhance Soybean Plant Growth and Aluminum Stress Tolerance by Shaping the Microbiome Assembly in an Acidic Soil

**DOI:** 10.1128/spectrum.03310-22

**Published:** 2023-03-14

**Authors:** Zhongling Wen, Minkai Yang, Hongwei Han, Aliya Fazal, Yonghui Liao, Ran Ren, Tongming Yin, Jinliang Qi, Shucun Sun, Guihua Lu, Shuijin Hu, Yonghua Yang

**Affiliations:** a Institute for Plant Molecular Biology, State Key Laboratory of Pharmaceutical Biotechnology, School of Life Sciences, Nanjing University, Nanjing, China; b Co-Innovation Center for Sustainable Forestry in Southern China, Nanjing Forestry University, Nanjing, China; c Jiangsu Key Laboratory for Eco-Agricultural Biotechnology around Hongze Lake, Huaiyin Normal University, Huai’an, China; d Department of Entomology & Plant Pathology, North Carolina State University, Raleigh, North Carolina, USA; Fujian Agriculture and Forestry University

**Keywords:** arbuscular mycorrhizal fungi (AMF), acidic soil, soybean, root-associated microbiome, Al stress tolerance

## Abstract

Strongly acidic soils are characterized by high aluminum (Al) toxicity and low phosphorus (P) availability, which suppress legume plant growth and nodule development. Arbuscular mycorrhizal fungi (AMF) stimulate rhizobia and enhance plant P uptake. However, it is unclear how this symbiotic soybean-AMF-rhizobial trio promotes soybean growth in acidic soils. We examined the effects of AMF and rhizobium addition on the growth of two soybean genotypes, namely, Al-tolerant and Al-sensitive soybeans as well as their associated bacterial and fungal communities in an acidic soil. With and without rhizobial addition, AMF significantly increased the fresh shoot and root biomass of Al-tolerant soybean by 47%/87% and 37%/24%, respectively. This increase in plant biomass corresponded to the enrichment of four plant growth-promoting rhizobacteria (PGPR) in the rhizospheric soil, namely, *Chitinophagaceae bacterium* 4GSH07, Paraburkholderia soli, Sinomonas atrocyanea, and Aquincola tertiaricarbonis. For Al-sensitive soybean, AMF addition increased the fresh shoot and root biomass by 112%/64% and 30%/217%, respectively, with/without rhizobial addition. Interestingly, this significant increase coincided with a decrease in the pathogenic fungus Nigrospora oryzae as well as an increase in *S. atrocyanea*, *A. tertiaricarbonis*, and Talaromyces verruculosus (a P-solubilizing fungus) in the rhizospheric soil. Lastly, the compartment niche along the soil-plant continuum shaped microbiome assembly, with pathogenic/saprotrophic microbes accumulating in the rhizospheric soil and PGPR related to nitrogen fixation or stress resistance (e.g., Rhizobium leguminosarum and Sphingomonas azotifigens) accumulating in the endospheric layer.

**IMPORTANCE** Taken together, this study examined the effects of arbuscular mycorrhizal fungi (AMF) and rhizobial combinations on the growth of Al-tolerant and Al-sensitive soybeans as well as their associated microbial communities in acidic soils and concluded that AMF enhances soybean growth and Al stress tolerance by recruiting PGPR and altering the root-associated microbiome assembly in a host-dependent manner. In the future, these findings will help us better understand the impacts of AMF on rhizosphere microbiome assembly and will contribute to the development of soybean breeding techniques for the comprehensive use of PGPR in sustainable agriculture.

## INTRODUCTION

Acidic soils occupy approximately 30% of the global land area and 50% of the potential cultivated land area in the world (1). Acidic red soil is characterized by a low pH value, low bioavailable phosphorus (P), and high Al toxicity. In many acidic soils, high Al^3+^ poses major problems for plant growth because it immobilizes soil P, damages root tips, and inhibits root elongation and absorption of water and nutrients, thereby reducing crop growth and yield (2–7).

The root-soil interface is a critical gateway for plants to take up water and nutrients from soils and exert their effects on soils, while root-soil interactions are very complex with a multitude of microbes actively participating in the association under natural conditions (8). The rhizosphere is not only a battlefield for plant roots resisting soilborne pathogens but also a playground for roots benefiting from rhizosphere beneficial microbes (9–11). Some soil microorganisms are important regulators of plant productivity, especially in low-fertility ecosystems, which can help symbiotic plants obtain limited nutrients. Rhizobia and arbuscular mycorrhizal fungi (AMF) are two of the most important groups of microbes that form symbiotic relations with plants. Symbiotic nitrogen fixation by rhizobia is an important source of nitrogen nutrition for plants, while AMF promote plant absorption of nutrients (such as P, N, Mg, and Ca) and improve plant resistance to environmental stresses (e.g., drought, heavy metals, and pathogens) (12–15).

In microscale environments like the rhizosphere, microbial communities are likely to be dominated by the microbial interactions that further affect the growth of host plants (16). For instance, rhizosphere microbiome members with growth-inhibitory siderophores can suppress some pathogens, while members with growth-promotive siderophores seem to facilitate plant infection due to inferior competition (17). *Phomopsis liquidambari* enriches the diversity of nodular culturable endophytic bacteria, while its mycelia have been found to be ideal dispersal networks for rhizobial enrichment in the legume rhizosphere soil, thereby improving the nitrogen nutrition of host plants (18, 19). Inoculating rhizobia has been shown to increase the relative abundance of phyla *Proteobacteria*, *Firmicutes*, and *Actinobacteria* that exhibit an aerobic-heterotrophic metabolism and play a crucial role in soil fertility (20). AMF can modify the assembly of rhizosphere or foliar microbial communities to promote rhizobia-legume symbiosis or improve plant growth (21, 22). A recent study further showed that *Rhizobium* spp. and AMF worked together in affecting rhizosphere bacteria, such as *Verrumicrobia*, *Proteobacteria*, *Gemmatimonadetes*, and *Firmicutes* to enhance the yield, seed size, and fatty acid content of soybean grown in a semiarid environment (23).

Soybean is the most important source of plant proteins and oil in the world. Soybeans commonly form symbiotic associations with both rhizobia and AMF, providing a unique system to examine the interactions among rhizobia, AMF, and indigenous microbes. Major soybean-producing regions are predominantly located in regions with acidic soils, which significantly suppress yields of many Al-sensitive soybeans (24). Even if the breeding of Al-tolerant cultivars is possible, strong soil acidity still constrains the growth of rhizobia and AMF and soybean yield potential (24–29). In addition, the tolerance of soybean to Al toxicity is closely related to root exudation, that is, root secretion of organic acids, such as citric acid and malic acid, that chelate Al (30). These exudates also affect soil microscale environments and the community composition of rhizosphere bacteria (31–33). Soybean rhizosphere soil has been reported to be robustly enriched with some plant growth-promoting rhizobacteria (PGPR), such as *Rhizobium* (34), while some symbiotic plant growth-promoting fungi (PGPF), such as mycorrhizal fungi, may constitute ideal dispersal networks in the legume rhizosphere soil to aid rhizobia enrichment (19, 35). However, how AMF and rhizobia affect the assembly of rhizosphere microbial communities and modulate host plant tolerance to Al stress is still poorly understood.

Using two soybean cultivars (Al sensitive and Al tolerant) as model hosts, we examined the impacts of rhizobia and AMF on rhizosphere microbes and their effects on soybean Al tolerance. Our overall hypothesis was that rhizobia and AMF improve soybean Al tolerance in acidic soils and reshape the rhizosphere microbiome composition and function. We anticipate that rhizobia and AMF will improve soybean tolerance by recruiting specific microorganisms. We also anticipate that these effects will be linked to host plant genotype.

## RESULTS

### Basic traits of plants and soils.

**FIG 1:**
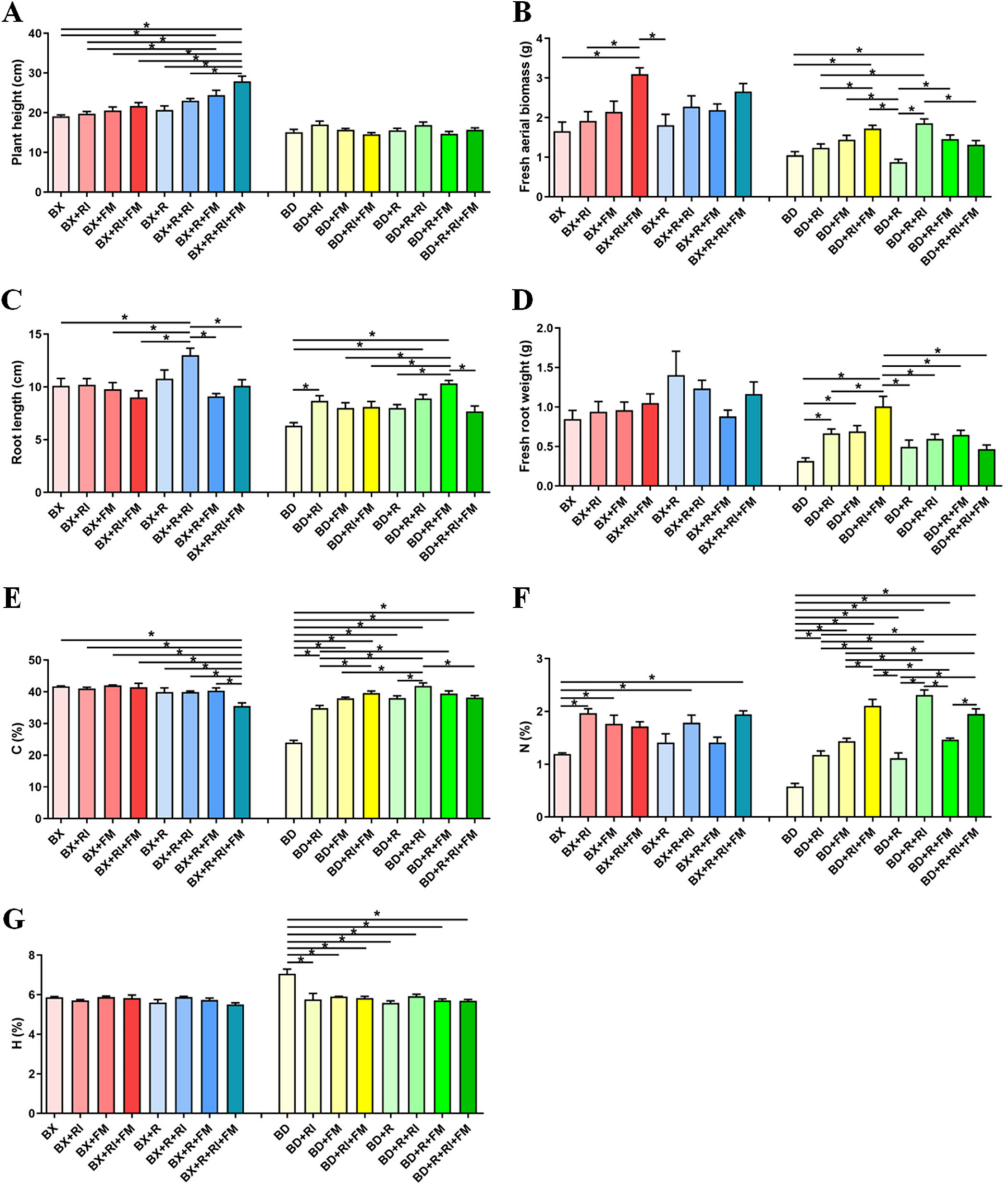
(A to G) Basic traits of plants. Plant height (A), fresh aerial biomass (B), root length (C), and fresh root weight (D) were measured as well as the carbon content (E), nitrogen content (F), and hydrogen content (G) of the aboveground part of the soybeans. BX10, BD2, R, RI, and FM represent the Al-tolerant soybean BX10, Al-sensitive soybean BD2, Ensifer fredii, *Rhizophagus intraradices*, and *Funneliformis mosseae*, respectively. Color in red/blue and yellow/green represent treatments of BX10 without/with rhizobium inoculation and of BD2 without/with rhizobium inoculation, respectively. The error bars represent the standard deviation of the plant samples. The asterisks indicate significant differences according to one-way ANOVA; *, *P* < 0.05.

Fig. 1 shows the basic phenotypic traits of plants, where *Rhizophagus intraradices* (RI) and *Funneliformis mosseae* (FM), two AMF, increased plant height and root length (Fig. 1A and C). With and without the addition of rhizobium, AMF boosted the aerial biomass of soybean in acidic soil by up to 47%/87% for Al-tolerant soybean Baxi 10 (BX10) and 112%/64% for Al-sensitive soybean Bendi 2 (BD2) (Fig. 1B). Similarly, AMF boosted root biomass by up to 37%/24% for BX10 and 30%/217% for BD2 with/without rhizobium application (Fig. 1D). AMF also significantly increased the C and N content of soybeans, particularly the Al-sensitive soybean BD2 (Fig. 1E and F). Inoculation with AMF and Ensifer fredii (R) decreased the H content of BD2 (Fig. 1G). Additionally, the acid phosphatase (S-ACP) activity of BD2 was significantly impacted by AMF addition, and the nitrite reductase (S-NiR) activity of BD2 was impacted by rhizobium application, whereas sucrase (S-SC), nitrate reductase (S-NR), and urease (S-UE) enzyme activities were unaffected (Fig. S1 in the supplemental material).

### Alpha diversity of soybean root-associated microbial communities.

**FIG 2:**
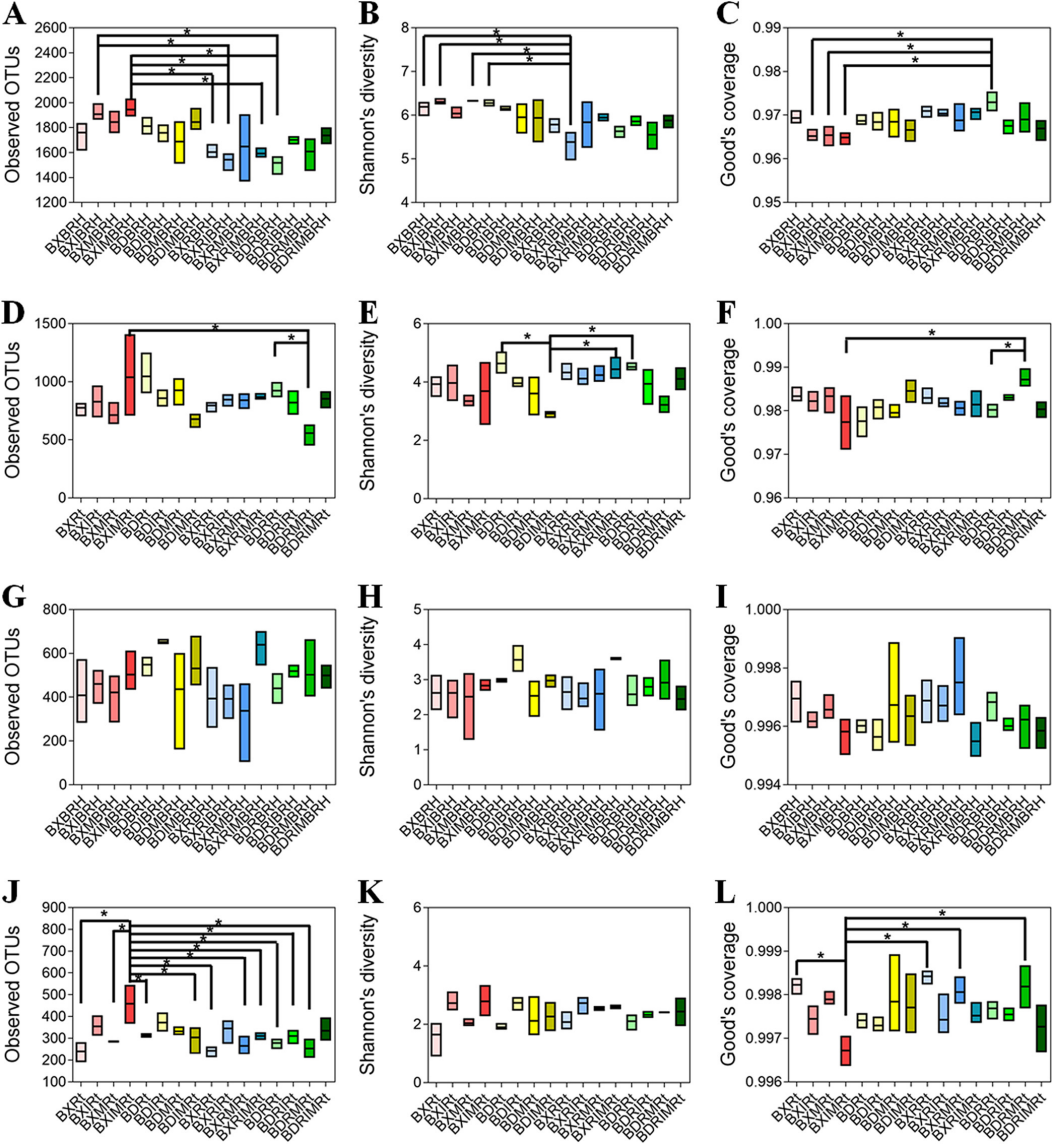
Box plots of alpha diversity of root-associated microbial communities. The results of alpha diversity analysis through three different indices (Observerd OTUs value, Shannon value, and Good’s coverage) were divided into four groups according to different host niches and sequencing results, including rhizospheric soil (A to C) and endospheric layer (D to F) samples of the bacterial community and rhizospheric soil (G to I) and endospheric layer (J to L) samples of the fungal community, respectively. These indices were analyzed by one-way ANOVA. BX, BD, R, I, M, BRH, and Rt represent aluminum-tolerant soybean BX10, aluminum-sensitive soybean BD2, Ensifer fredii, *Rhizophagus intraradices*, *Funneliformis mosseae*, rhizospheric soil, and endospheric layer, respectively. Color in red/blue and yellow/green represent treatment of BX10 without/with rhizobium inoculation and of BD2 without/with rhizobium inoculation, respectively. The asterisks indicate a significant difference according to one-way ANOVA; *, *P* < 0.05; OTUs, operational taxonomic units.

The addition of the rhizobium *E. fredii* reduced the bacterial community richness and diversity in rhizospheric soil, especially for the Al-tolerant soybean BX10 with *R. intraradices* inoculation (Fig. 2A to C). The coapplication of the AMF *R. intraradices* and *F. mosseae* in the endospheric layer (Rt) reduced the diversity of the bacterial community of the Al-sensitive soybean BD2 (Fig. 2D to F). As for the fungal community, the coapplication of AMF significantly increased the community richness and reduced the community coverage in the endospheric layer of BX10 (Fig. 2J to L). There was no substantial difference in fungal community richness, diversity, and coverage between different treatments in rhizospheric soil (Fig. 2G to I).

### Beta diversity of soybean root-associated microbial communities.

**FIG 3:**
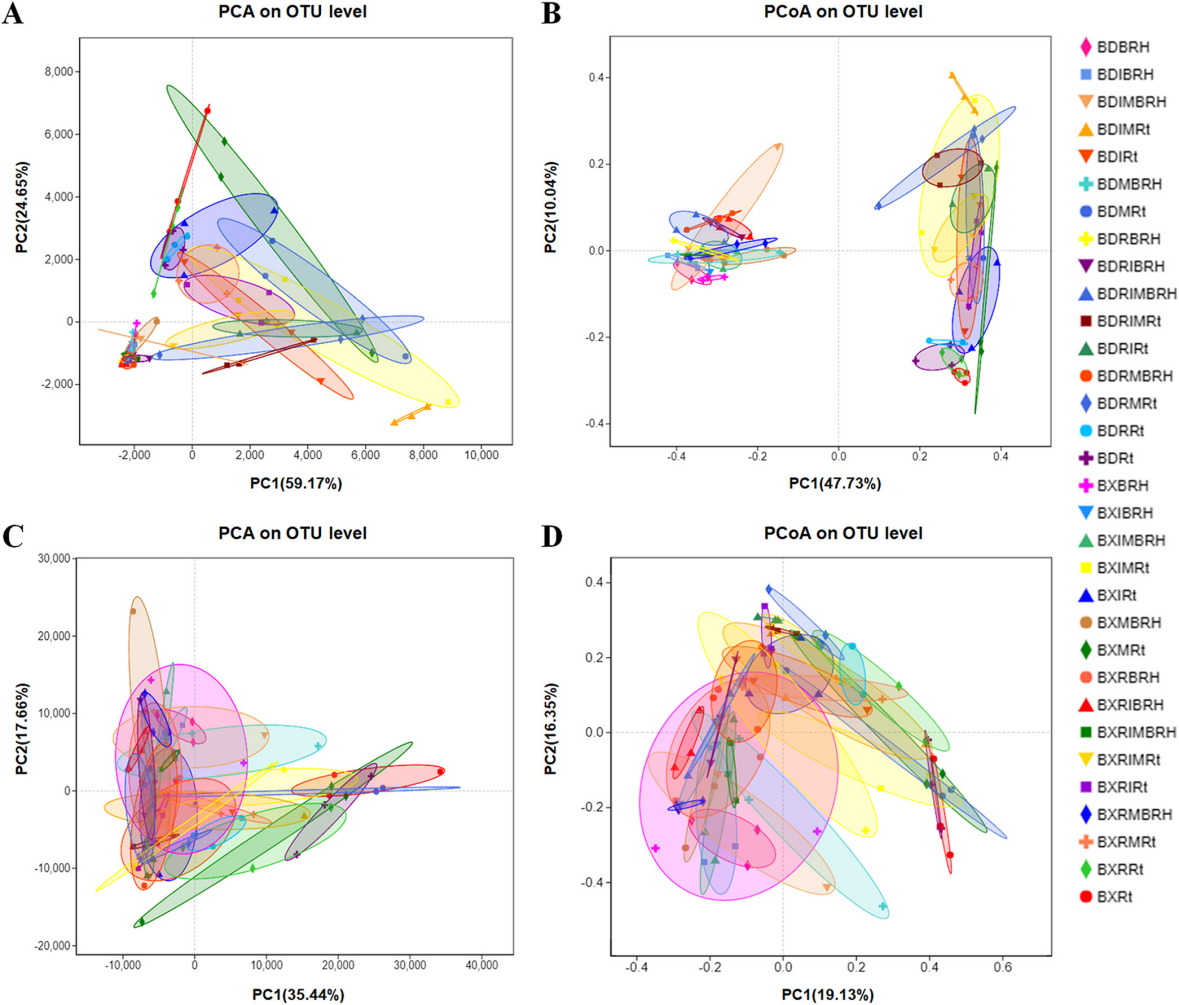
PCA and PCoA charts. (A) PCA based on the operational taxonomic unit (OTU) abundance of the bacterial community. (B) PCoA based on the Bray-Curtis distance of the bacterial community. (C) PCA based on the OTU abundance of the fungal community. (D) PCoA based on the Bray-Curtis distance of fungal community. The treatment details are shown in Fig. 2.

The principal-component analysis (PCA) and partial least-squares discriminant analysis (PLS-DA) chart revealed no significant difference in distance (Fig. 3A; Fig. S2A); however, the principal-coordinate analysis (PCoA) and nonmetric multidimensional scaling analysis (NMDS) chart revealed that the root-associated bacterial community was clearly segregated by these two different host niches (Fig. 3B; Fig. S2B). The PCA, PCoA, PLS-DA, and NMDS charts revealed no significant differences in the fungal community between the endospheric layer and the rhizospheric soil (Fig. 3C and D; Fig. S2C and 3D). The analysis of similarities (ANOSIM) and Adonis analyses of root-associated microbial communities showed that there was no difference between two soybean genotypes in the composition of root-associated bacterial and fungal communities (Table 1). However, statistical analysis revealed substantial variations in the microbial composition of the endospheric layer and rhizospheric soil, and AMF treatments also had an impact on the composition of microbial communities (*P* < 0.05) (Table 1; Table S1).

**TABLE 1:** Statistical analyses of root-associated microorganism community structure

Microorganism communities	Group versus group^*a*^	Adonis^*b*^^,^^*c*^		ANOSIM^*b*^^,^^*c*^	
		*R* ^2^	*P* value	Statistic	*P* value
Bacterial community	BX10 versus BD2	0.0296	0.026	0.0526	0.023
	BRH versus Rt	0.4280	**0.001**	0.9364	**0.001**
	NA_1_ versus RI	0.0619	0.028	0.1311	0.011
	NA_1_ versus FM	0.6832	**0.009**	0.116	0.014
	NA_1_ versus RI + FM	0.1074	**0.005**	0.2107	**0.002**
	RI versus FM	0.0199	0.371	−0.0008	0.355
	RI versus RI + FM	0.0340	0.142	0.0369	0.114
	FM versus RI + FM	0.0239	0.268	0.0058	0.277
	NA_2_ versus R	0.0553	**0.003**	0.1231	**0.002**
Fungal community	BX10 versus BD2	0.0150	0.178	0.0065	0.245
	BRH versus Rt	0.1727	**0.001**	0.4157	**0.001**
	NA_1_ versus RI	0.1219	**0.001**	0.254	**0.001**
	NA_1_ versus FM	0.0419	0.062	0.0486	0.061
	NA_1_ versus RI + FM	0.0593	0.011	0.0981	0.015
	RI versus FM	0.0467	0.024	0.067	0.017
	RI versus RI + FM	0.0462	0.024	0.0614	0.024
	FM versus RI + FM	0.0257	0.246	0.0119	0.267
	NA_2_ versus R	0.0934	**0.001**	0.2181	**0.001**

a NA_1_ represents no AMF treatment. NA_2_ represents no rhizobium treatment. BX10, BD2, R, RI, FM, BRH, and Rt represent Al-tolerant soybean BX10, Al-sensitive soybean BD2, Ensifer fredii, *Rhizophagus intraradices*, *Funneliformis mosseae*, rhizospheric soil, and endospheric layer, respectively.

b ANOSIM and Adonis based on the Bray-Curtis distance metric.

c The *P* values in bold indicate significant differences (*P* < 0.01) between groups.

### Comparison of the composition of the microbial taxa at different levels.

**FIG 4:**
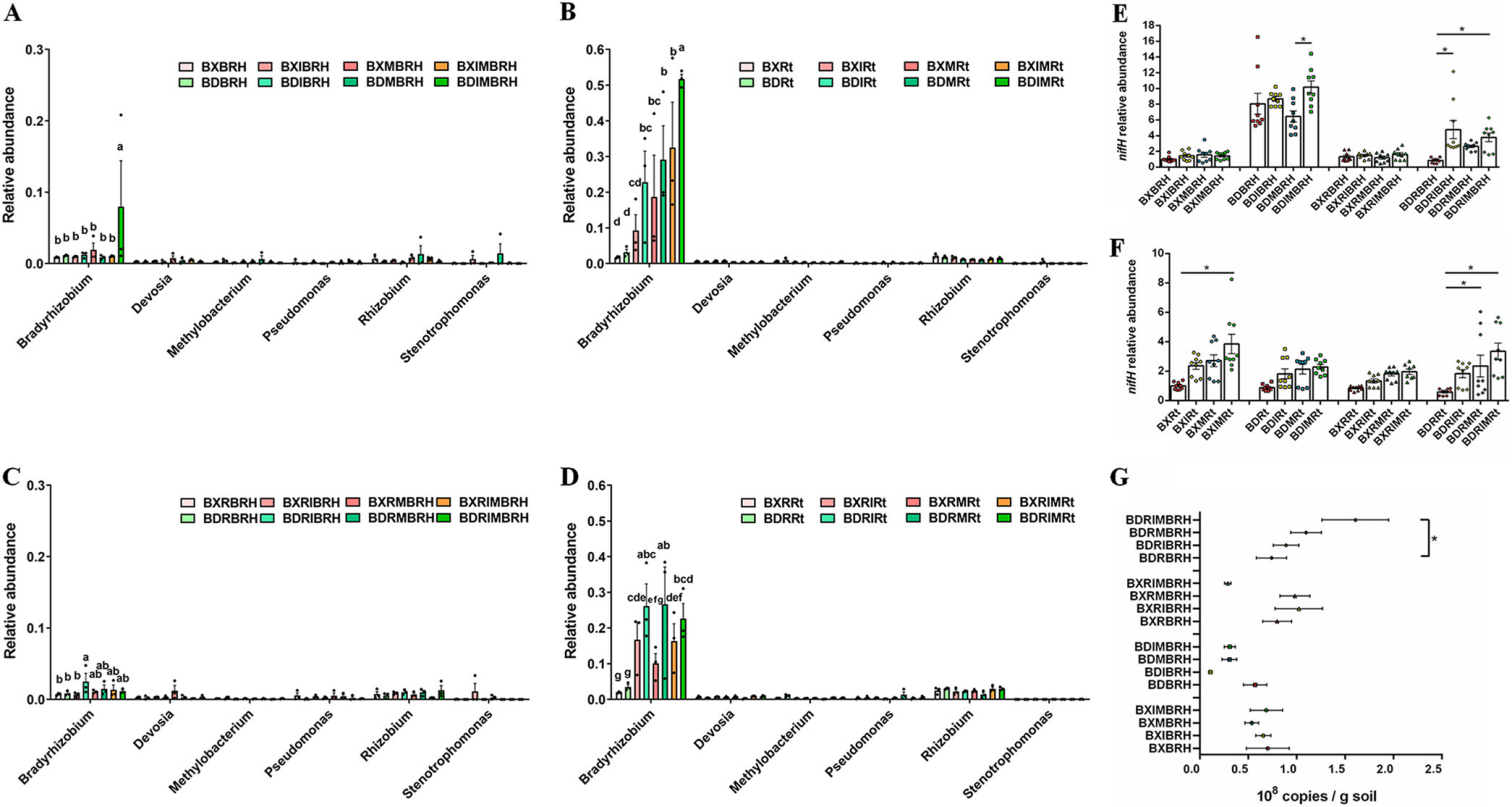
AMF affected the relative abundance of six genera related to nitrogen fixation, the relative abundance of the *nifH* gene, and the copy number of Fusarium. (A) The relative abundances of six nitrogen-fixing genera of samples without rhizobium treatment of rhizospheric soil samples. (B) The relative abundances of six nitrogen-fixing genera of samples without rhizobium treatment of endospheric layer samples. (C) The relative abundances of six nitrogen-fixing genera in rhizospheric soil samples with rhizobium treatment. (D) The relative abundances of six nitrogen-fixing genera in endospheric layer samples with rhizobium treatment. The error bars represent the standard deviation of three replicates of soil or root samples. Different superscript letters in A to D indicate significant differences (*P* < 0.01) between groups by one-way ANOVA. (E and F) The relative abundance of the *nifH* gene in rhizospheric soil samples (E) and endospheric layer samples (F) was determined by quantitative real-time PCR. Levels of *nifH* gene abundance were normalized to 16S rRNA gene abundance. A value of 1 was assigned to the detected value of rhizospheric soil of BX10. (G) Copies of Fusarium in rhizospheric soil at flowering stage were measured by absolute quantitative real-time PCR and also analyzed by one-way ANOVA. Error bars represent the standard deviation of three replicates of soil or root samples and each replicate with technical triplicate. Asterisks in E to G indicate significant differences between groups by one-way ANOVA; *, *P* < 0.01. The treatment details are shown in Fig. 2.

The comparison of the relative abundance of the top 11 major phyla of the bacterial community and the top eight major phyla of the fungal community (relative abundance of >1%) revealed that the addition of two AMF decreased the relative abundance of *Actinobacteria* and increased the relative abundance of *Proteobacteria* in the endospheric layer of Al-sensitive soybean BD2 (Table S2; Fig. S3). In addition, relative abundances of *Acidobacteria*, *Bacteroidetes*, *Chloroflexi*, *Firmicutes*, *Gemmatimonadetes*, *Mucoromycota*, *Rozellomycota*, and *Zoopagomycota* were significantly higher in rhizospheric soil than in endospheric layers, whereas *Proteobacteria* were significantly higher in the endospheric layer than in rhizospheric soil (Table S2; Fig. S3). Except for AMF, which enhanced the relative abundance of *Bradyrhizobium* in the endospheric layer of the Al-sensitive soybean BD2, there was no significant difference between different treatments in the relative abundance of six genera of bacteria related to nitrogen fixation (Fig. 4A to D). There was also no significant difference in the quantitative analysis of the relative abundance of the *nifH* gene in the soybean root-associated bacterial community (Fig. 4E and F) or the soybean root pathogenic fungus Fusarium (Fig. 4G).

Further comparison of the composition of all classified species showed that the application of rhizobium and AMF to Al-tolerant soybean BX10 increased the abundance of *Chitinophagaceae bacterium* 4GSH07 and Paraburkholderia soli in the rhizospheric soil. Additionally, AMF inoculation increased the abundance of Sinomonas atrocyanea and Aquincola tertiaricarbonis in the rhizospheric soil of both Al-tolerant and Al-sensitive soybeans (Table 2; Table S3). Moreover, AMF reduced the abundance of *Nigrospora oryzae* and Rhizopus microsporus in the rhizospheric soil of the Al-sensitive soybean BD2, but it increased the abundance of Aspergillus *foetidus* and *Talaromyces verruculosus* (Table 2; Table S3). Lastly, Sinomonas atrocyanea and Lacibacter cauensis were more abundant in the rhizospheric soil than in the endospheric layer, whereas the contrary was evident for *Chitinophagaceae bacterium* 4GSH07, Rhizobium leguminosarum bv. *viciae*, Sphingomonas azotifigens, Ralstonia pickettii, and Aquincola tertiaricarbonis (Table S3). No significant difference in fungal species abundance was observed between two different host niches (Table S3).

**TABLE 2:** Relative abundances of eight major bacterial and fungal species with different abundances among different rhizobium and AMF treatments

Species^*a*^	BXBRH	BDBRH	BXIBRH	BDIBRH	BXMBRH	BDMBRH	BXIMBRH	BDIMBRH
Sinomonas atrocyanea	0.0047644 ± 0.0015	0.0038667 ± 0.0007	0.0035215 ± 0.0005	0.0058001 ± 0.0019	0.0020887 ± 0.0008	0.003159 ± 0.0011	0.0027274 ± 0.0003	0.0030554 ± 0.0002
*Chitinophagaceae bacterium* 4GSH07	0.0006042 ± 0.0003	0.0004143 ± 0.0002	0.0001554 ± 0.0001	0.000397 ± 0.0004	0.000069 ± 0	0.0002417 ± 0.0002	0.0002244 ± 0.0002	0.0009322 ± 0.0006
Paraburkholderia soli	0.0068013 ± 0.003	0.0045572 ± 0.0033	0.0044191 ± 0.0023	0.0038322 ± 0.0021	0.0017262 ± 0.0016	0.0018125 ± 0.0012	0.0018471 ± 0.0004	0.003487 ± 0.0022
Aquincola tertiaricarbonis	0.0013119 ± 0.0018	0.0013982 ± 0.0011	0.0069567 ± 0.002	0.0023994 ± 0.0015	0.0092871 ± 0.0026	0.0028655 ± 0.0022	0.011652 ± 0.0052	0.0081996 ± 0.0021
Aspergillus foetidus	0.0025016 ± 0.0027	0.0033004 ± 0.0014	0.0005396 ± 0.0001	0.0036367 ± 0.0018	0.0003013 ± 0.0002	0.0030762 ± 0.0035	0.0005115 ± 0.0004	0.0727279 ± 0.1206
Talaromyces verruculosus	0.0001401 ± 0.0001	0.0001261 ± 0.0001	0.0000491 ± 0	0.0003714 ± 0.0002	0.000028 ± 0	0.0000631 ± 0.0001	0 ± 0	0.003882 ± 0.0064
Nigrospora oryzae	0.0016747 ± 0.0008	0.0066218 ± 0.0054	0.0014435 ± 0.0012	0.004856 ± 0.0035	0.0032023 ± 0.0026	0.0012963 ± 0.0014	0.0021232 ± 0.0013	0.0046528 ± 0.0048
Rhizopus microsporus	0.0084927 ± 0.0119	0.0225002 ± 0.0125	0.000021 ± 0	0.0003714 ± 0.0005	0.000007 ± 0	0.0003714 ± 0.0006	0.000007 ± 0	0.0000631 ± 0.0001
**Species**	**BXRBRH**	**BDRBRH**	**BXRIBRH**	**BDRIBRH**	**BXRMBRH**	**BDRMBRH**	**BXRIMBRH**	**BDRIMBRH**
Sinomonas atrocyanea	0.0093561 ± 0.0002	0.0112722 ± 0.0035	0.0110996 ± 0.0024	0.0112032 ± 0.0017	0.0054376 ± 0.0016	0.0058174 ± 0.0016	0.0118246 ± 0.0025	0.0127913 ± 0.001
Chitinophagaceae bacterium 4GSH07	0.0002417 ± 0.0002	0.0002589 ± 0.0001	0.0001381 ± 0.0002	0.0001381 ± 0.0001	0.0001899 ± 0.0001	0.0001726 ± 0.0001	0.0011393 ± 0.0007	0.0002071 ± 0.0001
Paraburkholderia soli	0.0070085 ± 0.0022	0.0071293 ± 0.0025	0.0032798 ± 0.0015	0.0033834 ± 0.0005	0.0030209 ± 0.0002	0.0059382 ± 0.0056	0.0150526 ± 0.0045	0.0050751 ± 0.0022
Aquincola tertiaricarbonis	0.0014328 ± 0.0004	0.0011048 ± 0.001	0.0047298 ± 0.0036	0.009943 ± 0.0023	0.0055066 ± 0.004	0.008165 ± 0.0049	0.0016744 ± 0.0005	0.0046953 ± 0.0005
Aspergillus foetidus	0.0008409 ± 0.0009	0.0024946 ± 0.0031	0.0004415 ± 0.0002	0.0008058 ± 0.0001	0.0017938 ± 0.0026	0.0018289 ± 0.0024	0.004863 ± 0.0022	0.000932 ± 0.0007
Talaromyces verruculosus	0.0001752 ± 0.0002	0.0001892 ± 0.0002	0.0000491 ± 0.0001	0.0006587 ± 0.0006	0.0001472 ± 0.0001	0.0000841 ± 0.0001	0.0001962 ± 0.0002	0.000035 ± 0
Nigrospora oryzae	0.0011912 ± 0.0009	0.0024735 ± 0.001	0.0007778 ± 0.0008	0.0006867 ± 0.0003	0.0007217 ± 0.0007	0.0006447 ± 0.0003	0.000932 ± 0.0003	0.0012823 ± 0.0008
Rhizopus microsporus	0.0122837 ± 0.018	0.0002172 ± 0.0002	0.000028 ± 0	0.000014 ± 0	0.0001962 ± 0.0002	0.000035 ± 0	0.0001121 ± 0	0.000042 ± 0.0001

a Statistical analyses was performed with a one-way ANOVA. The treatment details are shown in Table 1.

### Network complexity and functional prediction of microbial communities.

Microbial community Chao and Shannon index values were lower in the endospheric layer than in the rhizospheric soil (Fig. S4). In comparison to rhizospheric soil, the microbial community networks of the endospheric layer were less complicated (Fig. S4). The analysis of functional genes using PICRUSt showed that the composition and abundance of 16S rRNA functional genes exhibited no significant differences across all treatments, with amino acid transport and metabolism being the most prevalent functional genes, followed by energy production/conversion and signal transduction mechanisms (Fig. S5A and B). Fungi Functional Guild (FUNGuild) analysis of the fungal community revealed that one functional gene annotated as pathogen and saprotroph was higher in rhizospheric soil than in the endospheric layer (Fig. S5C).

A total of nine Clusters of Orthologous Groups (COG) functional classifications were found to be closely related to nitrogen fixation. Five of these, COG1348, COG2710, COG5420, COG5456, and COG5554, were shown to be enriched in the endospheric layer compared with the rhizospheric soil, and they were directly related to nitrogenase and nitrogen fixation protein (Table S4). In contrast, the relative abundance of COG1433 and COG3810, which are referred to as dinitrogenase iron-molybdenum cofactor biosynthesis protein and nitrogen regulatory protein, was higher in rhizospheric soil than in the endospheric layer (Table S4). Additionally, the relative abundance of COG0061, a catalyst for the phosphorylation of NAD to NADP, was considerably reduced by the coapplication of two AMF in the endospheric layer of the Al-sensitive soybean BD2 (Table S4).

## DISCUSSION

### Effects of AMF on soybean tolerance to acidic soils.

Our results showed that AMF improved soybean tolerance to acidic soils, likely by recruiting PGPR in the rooting zone, and increased plant biomass as well as carbon and nitrogen content (Table S3). Inoculation with AMF can improve the restoration of soil nitrogen-cycling microbial communities, which is closely related to nitrogen fixation (36). Interestingly, at the phylum, genus, and *nifH* gene levels of nitrogen-fixation bacteria, the results implied that AMF had no significant effect on the relative abundance of these bacteria (Fig. 4). Instead, AMF significantly increased the abundance of four species, including *C. bacterium*, *P. soli*, *S. atrocyanea*, and *A. tertiaricarbonis* (Table S3). These microbes have been previously identified as PGPR due to their ability to promote nitrogen fixation, plant disease resistance, and tertiary butyl moiety degradation (37–40). Meanwhile, AMF were also correlated with decreased levels of the plant pathogen *N. oryzae* (41) and enriched levels of the P-solubilizing fungus *T. verruculosus* (42) in the rhizospheric soil (Table S3). AMF can enhance the mineralization of soil organic P and positively affect root phosphatase activities (14, 43). In our investigation, both AMF and *T. verruculosus* enrichment appeared to operate synergistically in the P uptake of the host soybean. AMF inoculation has previously been reported to affect the composition and structure of the native plant microbiome, while biomass promotion was closely related to changes in the community composition of symbiosis-associated PGPR (44, 45). To better quantify these indirect effects of AMF, an in-depth understanding of the mechanisms of AMF is needed to emphasize the complex interactions that induce feedback between the microbiome and host plant as well as the functions of AMF (46, 47). Together, changes in the structure and abundance of microbial communities that promote PGPR microbes may enhance soybean plant tolerance to stress.

### Host dependence of AMF effects.

Compared to nontolerant cultivars, Al-tolerant soybean plants have better growth performance in acidic soils. However, most soybean cultivars with ideal yield and economic traits (e.g., high yield, high protein content, and herbicide resistance) do not have adequate Al stress tolerance, which restricts their ability to grow in acidic soils (48–50). Therefore, enhancing the resistance of the Al-sensitive soybean in acidic soils has undoubtedly huge agricultural implications. In the present study, it was discovered that AMF promoted aboveground plant biomass more in the Al-sensitive soybean than in the Al-tolerant soybean (Fig. 1). In addition, the presence of AMF increased the abundance of PGPR and a P-solubilizing fungus in rhizospheric soil of Al-sensitive soybean (Table S3), whereas it only increased PGPR in the rhizosphere of Al-tolerant soybean (Table S3). This difference may be due to the presence of distinct metabolic compounds in root exudates (51–53). Citrate, malate, and lactate in the soil have been found to have an ameliorative effect on the growth of soybean (48). Soybean plants release organic acids as root exudates to reduce Al toxicity in acidic soil (54). It has been reported that the Al-tolerant soybean BX10 has higher secretion rates of citric acid and malic acid than the Al-sensitive soybean BD2 (31, 55). We therefore speculate that the help of AMF resulted in greater improvements of host soybean growth (e.g., biomass) by changing the root-associated microbiome of the Al-sensitive soybean and recruiting P-solubilizing fungus due to the insufficient organic acid secretion of the Al-sensitive soybean BD2 in acidic soil, which led to the host dependence on AMF effects. Plants and AMF have trade-offs between dependence and benefit, and both benefit one other in mycorrhizal symbiosis to stabilize cooperation (56–58). Thus, the mechanisms behind AMF interactions, host plant organic acid secretion, and root-associated microbiome reassembly still need to be investigated further.

### The relationship between host niches of the soybean rhizosphere and microbiome assembly.

At the phylum level, some PGPR and humus-degrading bacteria were found to be enriched in rhizospheric soil (Table S2 and Fig. S3 in the supplemental material). At the species level, the soybean root (endospheric layer) was found to have positive selection (enrichment effect) for five bacterial species (e.g., *C. bacterium*, R. leguminosarum, and *S. azotifigens*), which belonged to the PGPR, rhizobia, metal-tolerant bacteria, and tertiary butyl moiety-degrading bacteria (Table S3) (37, 59–61). Other studies have shown that host selection pressure increased sequentially from soils to epiphytes to endophytes, reducing bacterial diversity and network complexity (62), resulting in differentiation of the structure and function of microbial communities between the endospheric layer and rhizospheric soil. Other studies have documented the hierarchical enrichment of the bacterial community from the rhizosphere to the rhizoplane to the endosphere in soybean (63). In addition, it has been reported that some PGPR epiphytes tend to form biofilms within an extracellular matrix, the roots provide a favorable attachment environment, and root-secreted organic acids can recruit beneficial soil bacteria (64), which may contribute to the augmented effects of these PGPR in the endospheric layer of soybean. Some PGPR, such as rhizobia, are part of the indigenous enrichment microbiome of the soybean rhizosphere and help soybean in the absorption of nutrients in disturbed or infertile soils (34, 64). Therefore, in addition to the synergistic effect of AMF, the host selection effect also aids plants in accumulating PGPR and achieving better growth and yield in acidic Al stress environments. One major limitation of our study was that only vegetative traits were assessed. Future studies should examine whether and how the effects of AMF on vegetative growth affect soybean yield traits, particularly protein and lipid contents.

### Conclusions.

In summary, our results showed that AMF increased soybean tolerance to acidic soils by recruiting special PGPR, while the host dependence of AMF effects was manifested specifically by enriching P-solubilizing fungus and decreasing the abundance of pathogenic fungus in the rhizospheric soil of Al-sensitive soybean. The host niches of the soybean rhizosphere also significantly affected the root-associated microbiome by lowering pathogenic and saprotrophic microbes and enriching PGPR in the endospheric layer. Together, our results show that AMF, host plant genotypes, and host niches coregulate the fine-tuning of microbial profiles and favor root-associated microbial communities to enhance the resistance of Al-tolerant soybean to acidic soil stress. Our findings suggest that plant breeding needs to consider the roles of complex rhizosphere microbiome interactions in the tolerance of host plants to environmental stresses such as Al toxicity and acidity.

## MATERIALS AND METHODS

### Plants, AMF and rhizobium strains, and soil materials.

In this study, the Al-tolerant soybean BX10 (Baxi 10) and the Al-sensitive soybean BD2 (Bendi 2) were used as model plants. *Rhizophagus intraradices* (RI; formerly *Glomus intraradices* [GI]) and *Funneliformis mosseae* (FM; formerly *Glomus mosseae* [GM]) were provided by the Institute of Plant Nutrition and Resources, Beijing Academy of Agriculture and Forestry Sciences (Beijing, China). The national microbial resources platform number of GI is 1511C0001BGCAM0062, while the number of GM is 1511C0001BGCAM0042. A rhizobium treatment was set up to eliminate the possible N stress in acidic soil. The rhizobium Ensifer fredii (Sinorhizobium fredii; R) was purchased from the Guangdong Microbial Culture Center (GDMCC), and the ATCC number is ATCC 35423. The acidic red soil was collected from the Ecological Experiment Station of Red Soil (28.208°N, 116.937°E) of the Chinese Academy of Sciences in Yingtan, Jiangxi Province, China (55, 65). The soil had a pH of 4.54 (±0.21) and a water holding capacity of 29.3%.

### Planting and sampling methods.

Before planting, a total of 10 g of AMF (10 g of a single species or 5 g of each species in a mixture) inoculum was added to the soil (~2 cm below the surface of the soil). Soybean seeds were disinfected with chlorine for 16 h, washed with sterile water 3 to 5 times, disinfected with 70% ethanol for 30 s and with 2.5% sodium hypochlorite for 5 min, and finally washed with sterile water 3 to 5 times. A diluted rhizobium culture solution was treated with seed dressing for 24 h, and an additional 50 mL of rhizobium culture solution was applied on the 10th and 15th days after planting. The details of planting and watering were described in previous studies (55, 66, 67). Each of the 32 treatments had three biological replicates. At the flowering stage, the plants were carefully excavated from the soil, avoiding any roots that were at the interface between the plot and the soil. The plant and soil materials were placed in a plastic bag with several prefreezing chemical ice packs and then immediately taken to the laboratory (55, 68, 69). As one of the two host niches of the soybean rhizosphere, rhizospheric soil samples were collected by brushing off the soil that was tightly adhered to the root surface with phosphate-buffered saline (PBS) and named BRH. The roots were then further washed with PBS twice, followed by centrifuging at 4,000 × *g*. Root samples, as another host niche, were collected by grinding with liquid nitrogen after being washed with PBS, which was defined as the endospheric layer (Rt). The soil and root samples were then stored at −80°C in a freezer before DNA extraction.

### Determination of soil enzyme activities.

Six key enzymes that are involved in the C, N, and P cycles of the root-associated microbial community were measured by corresponding kits purchased from Solarbi, China, including nitrate reductase (S-NR; EC 1.7.99.4), nitrite reductase (S-NiR; EC 1.7.99.3), urease (S-UE; EC 3.5.1.5), sucrase (S-SC; EC 3.2.1.26), acid phosphatase (S-ACP; EC 3.1.3.2), and alkaline phosphatase (S-AKP/ALP; EC 3.1.3.1). All the rhizospheric soil used to detect enzyme activities was filtered through a 50-μm mesh sieve after natural air drying. The determination steps were performed according to the detailed instructions.

### DNA extraction from soil and root samples.

In this study, all the DNA extraction methods were used as described by previous studies with minor modifications (55, 70, 71). Metagenomic DNA was extracted from approximately 0.30 g of rhizospheric soil or 0.40 g of root powder by using a PowerSoil DNA isolation kit (MoBio Laboratories, Inc., Carlsbad, CA, USA) after grinding with a tissue grinder (Grinder-48, Gallop Technology) at 60 Hz for 600 s. The DNA samples were assessed for quality on a 1% agarose gel and quantified by using a Qubit fluorometer (Qubit 2.0, Invitrogen, Carlsbad, CA, USA) (72). The concentration of each metagenomic DNA sample was tested and ensured to be more than 10 ng/μL.

### DNA amplicon sequencing and clean data.

Amplicons of approximately 468 bp encompassing the V3-V4 hypervariable region of the 16S rRNA were obtained by using the following primers: forward primer 338F (5′-ACTCCTACGGGAGGCAGCAG-3′) and reverse primer 806R (5′-GGACTACHVGGGTWTCTAAT-3′) (73). Additionally, amplicons of approximately 350 bp encompassing the internal transcribed spacer (*ITS1* region) were amplified using the following primers: forward primer ITS1F (5′-CTTGGTCATTTAGAGGAAGTAA-3′) and reverse primer ITS2R (5′-GCTGCGTTCTTCATCGATGC-3′) (74). PCR amplification, product purification, library quality determination, and high-throughput sequencing of the qualified libraries on the Illumina MiSeq platform (Illumina, CA, USA) with the MiSeq reagent kit were conducted by Majorbio Bio-pharm Technology, Co., Ltd. (Shanghai, China).

### Alpha diversity, beta diversity, and functional analyses.

The databases silva128/16s (http://www.arb-silva.de) and Unite8.0/ITS_fungi (http://unite.ut.ee/index.php) were used in this study, and the sequencing results were subsampled according to the minimum sample number of each. Alpha diversity analysis was used to reflect the community richness, diversity, and coverage, while beta diversity analysis was used to evaluate differences in species complexity (75, 76). The Observerd OTUs index, Chao index, Shannon index, and coverage index were used to assess alpha diversity, while beta diversity was analyzed through principal-component analysis (PCA), principal-coordinate analysis (PCoA), nonmetric multidimensional scaling analysis (NMDS), and partial least-squares discriminant analysis (PLS-DA). The analysis of functional genes in root-associated microbial communities was conducted by using the software PICRUSt and the Fungi Functional Guild (FUNGuild). All these analyses were performed on the platform I-Sanger (https://cloud.majorbio.com) as described previously (36, 77).

### Quantification of *nifH* by quantitative real-time PCR (qPCR).

To compare the relative abundance of the *nifH* gene in different samples, a quantitative real-time PCR was conducted by using the primer pairs of PolF-PolR and 338f-518r (78). The final 20-μL reaction volume of qPCR contained 50 ng of metagenomic DNA (~1 μL), 0.5 μL of each primer (5 pM), and 10 μL of 2× SYBR green mixture (FastStart universal probe master, Roche Life Science, Switzerland). The PCR program was as follows: 95°C for 10 min followed by 40 cycles consisting of 15 s at 95°C and 1 min at 60°C (70). The relative gene level was calculated by using the cycling threshold (2^−Δ^*^CT^*) method (79). All qPCRs were run in three technical duplicates with each DNA sample, and each treatment group contained three biological replicates.

### Statistical analyses.

A one-way analysis of variance (ANOVA) was used to evaluate the statistical analyses of plant and nutrient characteristics, enzyme activities, alpha diversity indices, quantification of the *nifH* gene, and comparison of the composition of the microbial taxa at different levels by using GraphPad Prism 8 software. The analysis of similarities (ANOSIM) and Adonis were performed by using the vegan package in R (v3.1.3) based on the Bray-Curtis distance metric (80).

### Data availability.

All clean sequencing data associated with this study are deposited in the Sequence Read Archive (SRA), and the accession numbers are PRJNA686490 and PRJNA686500.
