# Supplementary material for: Mycorrhizae Enhance Soybean Plant Growth and Aluminum Stress Tolerance by Shaping the Microbiome Assembly in an Acidic Soil
Source: Microbiol Spectr. 2023 Mar 14;11(2):e03310-22. doi: 10.1128/spectrum.03310-22 (PMC10100836; doi:10.1128/spectrum.03310-22)
Supplement: Supplemental file 2 — Fig. S1 to S5. Download spectrum.03310-22-s0002.pdf, PDF file, 1.1 MB [file spectrum.03310-22-s0002.pdf]

Mycorrhizae enhance soybean plant growth and Al stress tolerance by shaping the microbiome assembly in an acidic soil

Supplementary Figures

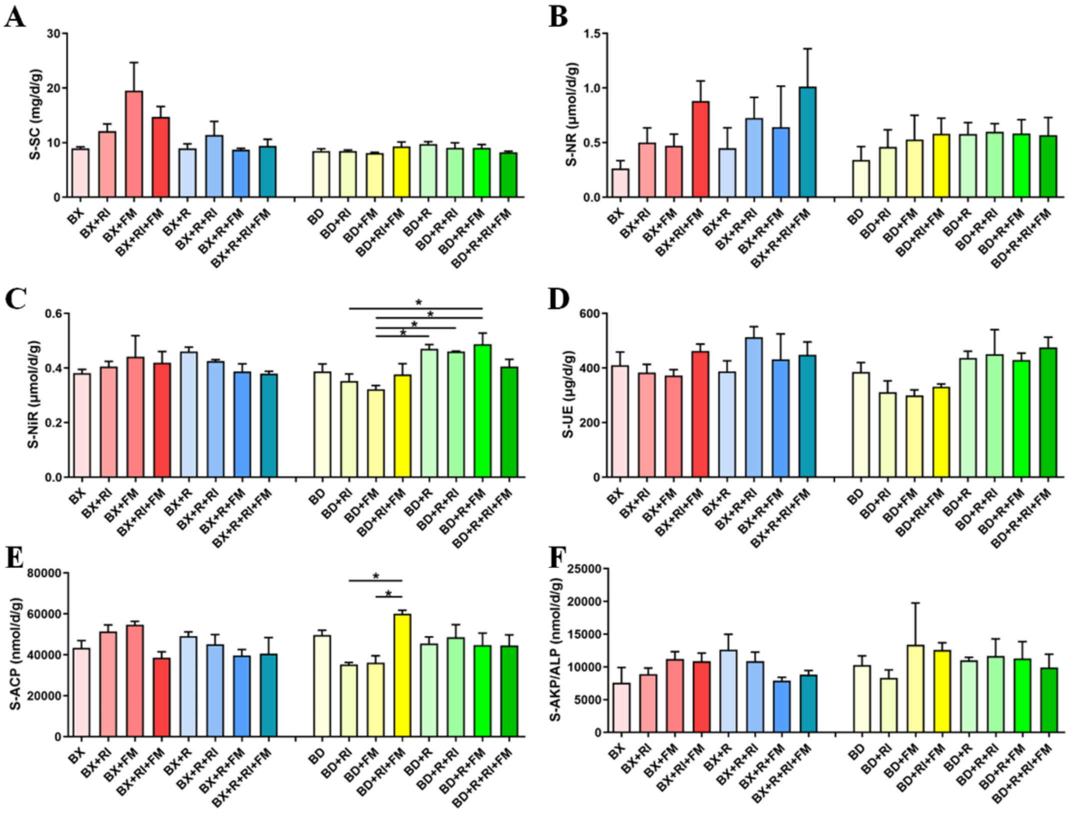

Supplementary Fig 1 The activities of six key enzymes involved in the carbon, nitrogen and phosphorus cycle of root-associated microbial communities. The activities of sucrase (A), nitrate reductase (B), nitrite reductase (C), urease (D), acid phosphatase (E) and alkaline phosphatase (F) in rhizospheric soil were analyzed by using one-way ANOVA. BX, BD, R, RI and FM represent Al-tolerant soybean BX10, Al-sensitive soybean BD2, *Sinorhizobium fredii*, *Rhizophagus intraradices* and *Funneliformis mosseae*, respectively. Color in red/blue and yellow/green represent treatments BX10 without/with rhizobium inoculation and BD2 without/with rhizobium inoculation, respectively. The error bars represent the standard deviation of the soil samples. The asterisk (*$p < 0.05$) indicates a significant difference according to one-way ANOVA.

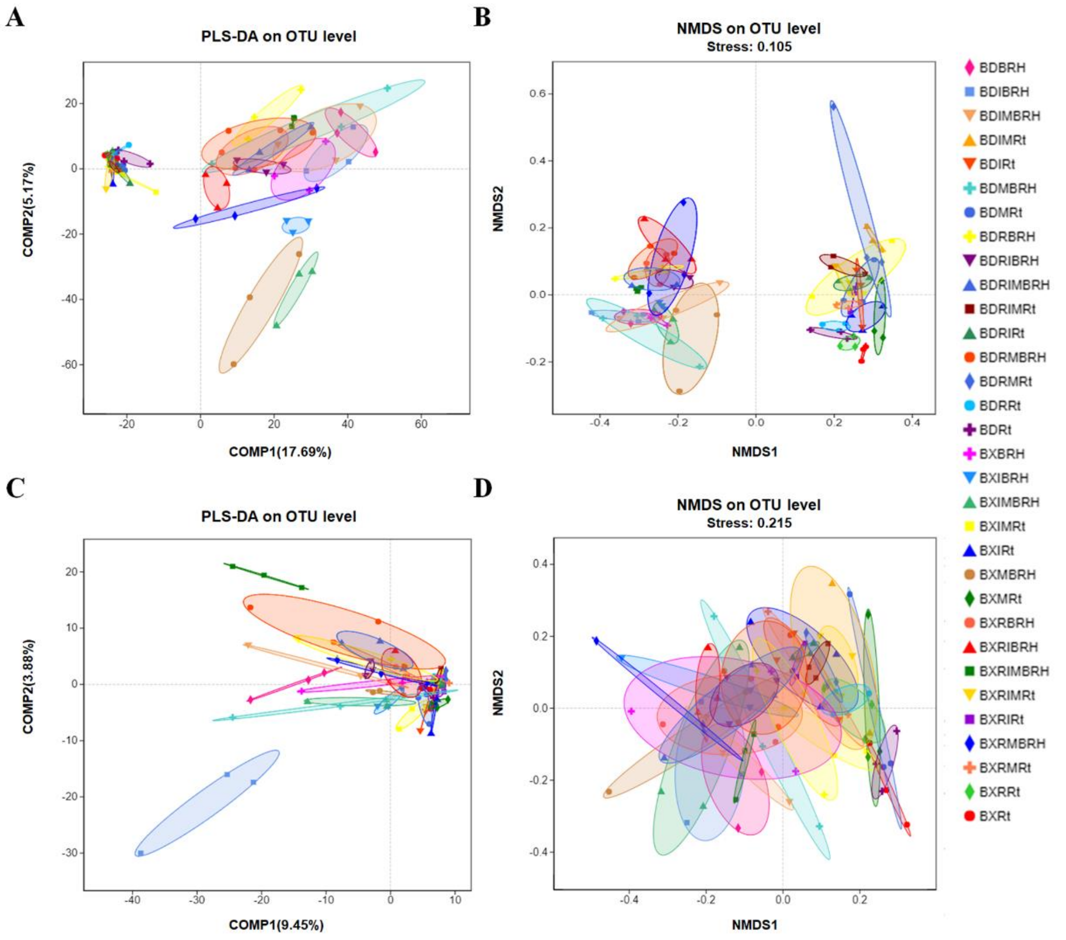

Supplementary Fig 2 PLS-DA and NMDS charts. PLS-DA (A) and NMDS (B) charts of the bacterial community. PLS-DA (C) and NMDS (D) charts of the fungal community. BX, BD, R, I, M, BRH and Rt represent aluminum tolerant soybean BX10, aluminum sensitive soybean BD2, *Sinorhizobium fredii*, *Rhizophagus intraradices*, *Funneliformis mosseae*, rhizospheric soil and endospheric layer, respectively.

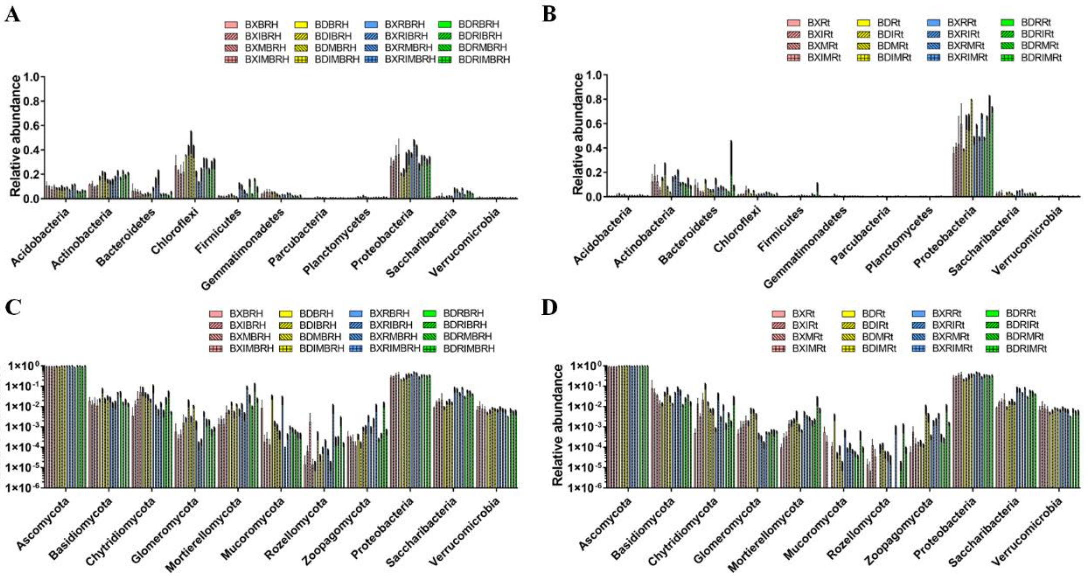

Supplementary Fig 3 Relative abundances of major phyla of the microbial communities in each sample with abundances higher than 1%. (A). Top eleven major phyla of the bacterial community of rhizospheric soil samples. (B). Top eleven major phyla of the bacterial community of endospheric layer samples. (C)Top eight major phyla of the fungal community of rhizospheric soil samples. (D). Top eight major phyla of the fungal community of endospheric layer samples. Color in red/blue and yellow/green represent treatments BX10 without/with rhizobium inoculation and BD2 without/with rhizobium inoculation, respectively. The error bars represent the standard deviation of the soil samples. The treatment details were shown in Supplementary Fig. 2.

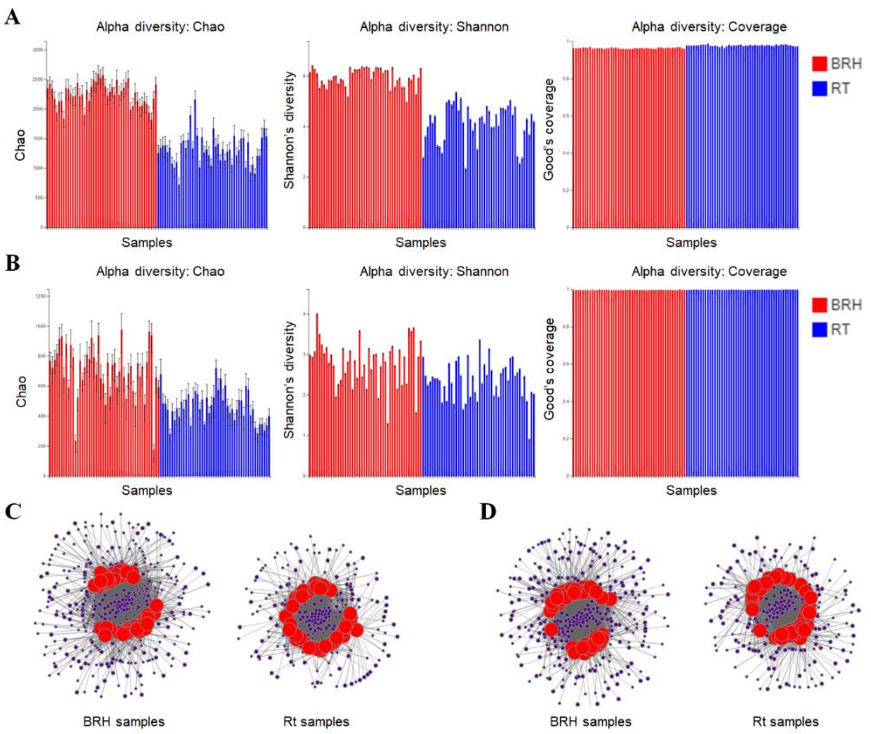

Supplementary Fig 4 Host selection reduced the richness, diversity and network complexity of root-associated bacterial communities. The results of alpha diversity through three different indices (Chao value, Shannon value and Good's coverage) of different host niches from 16S rRNA sequencing results (A) and ITS sequencing results (B). Network analysis of BRH and Rt samples were drawn by software Networkx according to 16S rRNA sequencing results (C) and ITS sequencing results (D). BRH and Rt represent rhizospheric soil and endospheric layer, respectively.

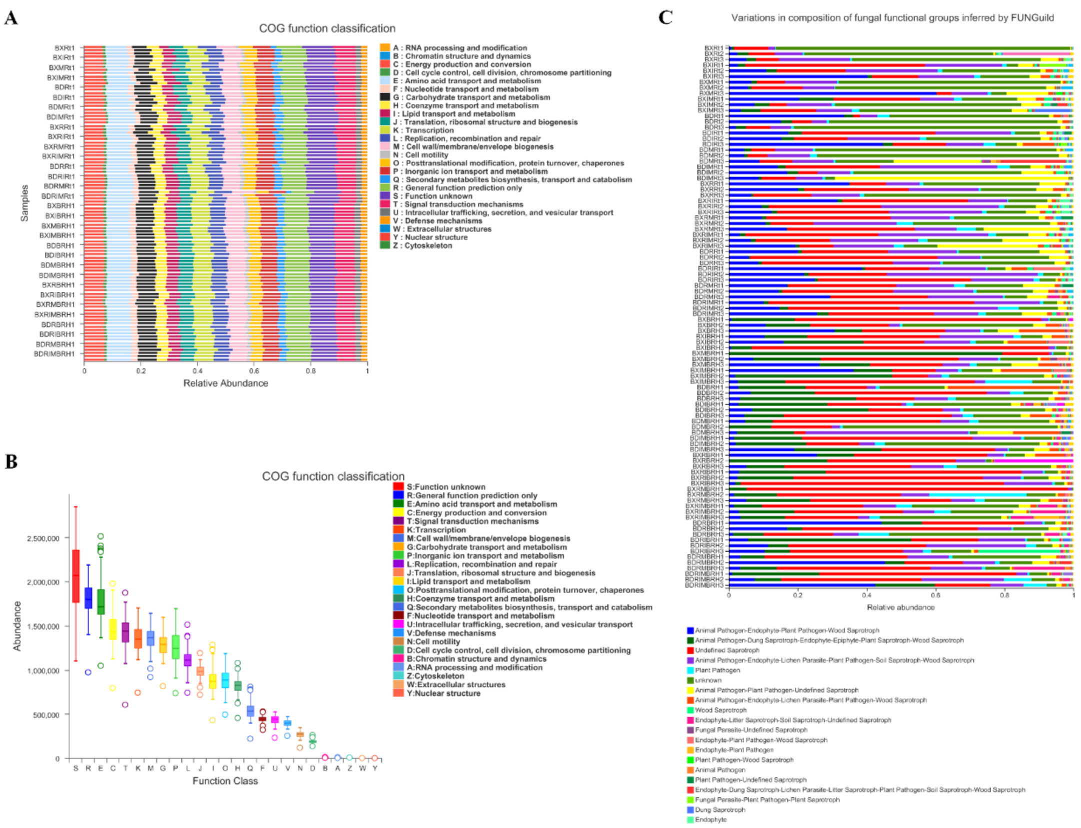

Supplementary Fig 5 Composition and abundance of functional genes in the root-associated microbial communities. (A). Composition and abundance of functional genes in the bacterial community. (B). Box-plot of 16S rRNA functional gene abundance. (C). Composition and abundance of functional genes in the fungal community. The treatment details were shown in Supplementary Fig. 2.
